# The effect of different traditional Chinese exercises on bone mineral density in menopausal women: a systematic review and network meta-analysis

**DOI:** 10.3389/fpubh.2024.1430608

**Published:** 2024-09-12

**Authors:** Jun Hou, Haiping Mao, Peiyao Xie, Yuemei Cui, Ming Rong

**Affiliations:** ^1^Faculty of Sports Science, Ningbo University, Ningbo, China; ^2^Research Academy of Grand Health, Ningbo University, Ningbo, China

**Keywords:** traditional Chinese exercises, menopausal, bone mineral density, network meta-analysis, meta-analysis

## Abstract

**Objective:**

To evaluate the optimal choice of traditional Chinese exercise (TCE) for improving bone mineral density in postmenopausal women through a network meta-analysis.

**Methods:**

The Chinese and English databases were searched, including China National Knowledge Infrastructure, Chongqing VIP, PubMed, Web of Science and Cochrane Library. The outcomes included BMD of lumbar L2-L4, femoral neck, ward triangle, and greater trochanter. Standardized mean differences (SMD) and 95% confidence intervals (CI) were used to assess the correlation between each group of interventions, and surface under the cumulative ranking (SUCRA) was used to rank the best interventions.

**Results:**

A total of 20 randomized controlled trials (RCTs) including 1933 subjects and six interventions: [Tai Chi (TC), Baduanjin (BDJ), Wuqinxi (WQX), Yijinjing (YJJ), TA (Tai chi plus calcium supplementation), BA (Baduanjin plus calcium supplementation)] and three control measures: [Calcium supplementation (CA), Aerobic exercise (AE), Not exercise intervention (NEI)] were analyzed. Regarding BMD of lumbar L2-L4: TC [SMD = 0.99 (0.62, 1.37)], BDJ [SMD = 2.12 (1.39, 2.85)], TA [SMD = 1.12(0.17, 2.07)], BA [SMD = 2.24 (1.16, 3.31)] were superior in increasing BMD of lumbar L2-L4 relative to NEI. Regarding BMD of femoral neck: TC [SMD = 1.24 (0.70, 1.78)], BA [SMD = 3.77 (1.98, 5.56)] were superior in increasing BMD of femoral neck relative to NEI. Regarding BMD of ward’s triangle: TC [SMD = 1.63 (1.09, 2.17)] was superior in increasing BMD of ward’s triangle relative to NEI. Regarding BMD of greater trochanter: TC [SMD = 0.98 (0.28, 1.68)] were superior in increasing BMD of greater trochanter relative to NEI. TC topped the SUCRA with BMD of lumbar L2-L4: 53.8, femoral neck: 74.9, ward’s triangle: 86.9 and greater trochanter: 77.7.

**Conclusion:**

Four TCE (TC, BDJ, TA and BA) are all effective in partially improving BMD indicators in postmenopausal women, while TC was effective on all four BMD indicators, which seems to be recommended as the most suitable exercise modality for postmenopausal women.

**Systematic review registration:**

This research follows the PRISMA Network statement. The protocol for this study has been registered in the International Prospective Registry of Systematic Reviews (PROSPERO). CRD42023414944.

## 1 Introduction

Post-menopause is the period after the permanent cessation of a woman’s menstrual cycle, during which a woman’s bone health is threatened due to reduced and stopped production of estrogen from the ovary (1). Bone mineral density (BMD) declines rapidly during the menopausal transition and continues to decline after menopause. According to a cohort study involving 1,038 women (2), the BMD of Lumbar and femoral neck decreased to 0.006 and 0.004 g/cm2, respectively, for each additional year after the last menstrual period. The 10-year cumulative loss of BMD was 10.6% in the lumbar spine and 9.1% in the femoral neck (1). Low bone mineral density is one of the most important determinants of fracture risk (3). Osteoporosis or low bone mass is reported in 30–40% of postmenopausal women (4–6) and more than 30% of patients experienced at least one fracture (7, 8). Almost all fractures are associated with an increased risk of premature death (9). The researchers found a 2.43-fold and 1.82-fold increase in mortality from hip and vertebral fractures among community-living older women, respectively (10). Therefore, effective interventions to prevent and reduce bone loss in postmenopausal women is necessary.

Bone is a dynamic tissue with the ability to reshape its material and structural properties to accommodate mechanical loading (11). Increased load stimulation and vigorous muscular activity can increase bone mass and promote bone health (12). Therefore, postmenopausal women are advised to exercise to maintain bone mass or slow bone loss (13, 14). Studies have found that high-intensity and high-weight-bearing exercise is beneficial to increase bone density in postmenopausal women (15–17). However, for reasons of safety and operability, it is difficult to implement such an exercise pattern for postmenopausal women.

Chinese traditional sports are becoming popular all over the world. Tai Chi is light and soft; It combines motion and static, emphasizes the control of respiratory rhythm with consciousness, these exercises work the muscle groups in the core of the trunk. Therefore, long-term TC exercise produced stress changes in the lumbar spine and femur, which affected BMD at these sites. The eight movements of BDJ are simple and slow, mainly based on isometric contractions of muscles. It mainly emphasizes the training of the muscle strength of the lower limbs to improve the balance ability of the lower limbs and reduce the occurrence of fractures. In addition, BDJ increases bone pressure, promotes bone formation, reduces bone resorption, and controls the progression of osteoporosis. In addition, WQX is imitation five animals invented a kind of simple and safe. Movements involve the body’s main muscle groups and spine, limb, finger joint movement, can improve the blood circulation of the spinal joints, such as soft tissue, help maintain the normal structure of bones and joints, delay the development of osteoporosis. Meanwhile, YJJ emphasizes strengthening muscle and bone. Long-term exercise can improve the flexibility of muscles and ligaments, increase bone mineral density, and prevent and treat diseases (18). Traditional Chinese exercises (TCEs) such as Tai Chi (TC), Baduanjin (BDJ), Wuqinxi (WQX) and Yijinjing (YJJ) are very safe exercises for postmenopausal women. It is also recommended for the treatment or prevention of osteoporosis in middle-aged and older adult people (19).

Recently, Zhang et al. (20) conducted a network meta-analysis and found that mind–body exercises (such as TC, yoga, dance, and WQX) may be the best type of exercise to increase bone density in the lumbar spine and femoral neck of patients with osteoporosis and osteopenia. Two systematic reviews (SRs) of TCEs on bone mineral density in postmenopausal women have been published in 2016 and 2017 (21, 22), but their conclusions are contradictory. Recently, studies on the effect of TCEs on bone mineral density of menopausal women mainly focus on exploring the effect of a certain kind of traditional exercise on bone mineral density of menopausal women, while there are few studies comparing which kind of traditional exercise among two or more kinds of traditional exercise has better effect on bone mineral density of menopausal women. Network meta-analysis (NMA) can overcome this limitation by drawing together direct and indirect comparisons of all available treatment options. Therefore, the aim of this study was to conduct a systematic review and network meta-analysis comparing the effects of TC, WQX, YJJ, BDJ, and exercise plus calcium intervention on the BMD of lumbar L2–L4, femoral neck, ward’s triangle and greater trochanter in postmenopausal women and to examine whether one protocol is better than the others.

## 2 Materials and methods

### 2.1 Registration

This research follows the PRISMA Network statement. The protocol for this study has been registered in the International Prospective Registry of Systematic Reviews (PROSPERO). The registration number is CRD42023414944.

### 2.2 Literature search strategy

The search strategy was created by a combination of medical subject heading (MeSH) terms, keyword, and phrases. A comprehensive search strategy was finished and applied to the following databases in the first week of February 2023 with no date restriction: China National Knowledge Infrastructure (CNKI), Chongqing VIP, PubMed, Web of Science and Cochrane Library databases. The search term “((traditional Chinese exercise) OR (TC) OR (baduanjin) OR (wuqinxi) OR (yijinjing) and (osteoporosis) OR (bone loss) OR (bone mineral density) OR (bone metabolism) OR (bone density)), and all the results of these databases were imported to the software EndNote X9.1. After excluded duplicates, review, systematic review, meta-analysis, designs and protocols are excluded through the limitation of subject words. Then two researchers (H.J. and C.Y.) screened all titles and abstracts. Once the abstract suggested that these studies were potentially appropriate, the full-text were screened, and then it is included if the article meets the inclusion and exclusion criteria. In case of disagreement between the first two reviewers, a third reviewer (R.M.) was consulted.

### 2.3 Selection criteria

#### 2.3.1 Inclusion criteria

According to participants, interventions, comparisons, outcomes, and study design (PRICOS), the inclusion criteria strategy was defined. (1) Subjects aged 45 years old and over or postmenopausal woman were included. (2) The type of study included was RCT comparing the effect of different TCEs on bone mineral density. Experimental groups adopted TC, BDJ, YJJ, WQX, BA and TA. The control groups adopted non-exercise intervention, Ca, AE. (3) The studies outcomes at least one of the four BMD indicators (lumbar spine (L2-L4); Femoral neck; Femoral head greater trochanteric; Ward’s Triangle) should be included. (4) The study design was ethically approved and a RCTs.

#### 2.3.2 Exclusion criteria

The exclusion criteria included: (1) literature not published in Chinese or English. (2) duplicate published literature. (3) review and theoretical literature. (4) literature with only abstracts but no full text. (5) literature designed for non-RCT, for example self - control experiments before and after. (6) the experimental subjects were not>45 years old or women who explicitly state that they are not postmenopausal and so on. (7) the experimental data were not meet inclusion criteria or not clear, and it was not possible to calculate the average and standard deviation of the outcome indicator. (8) studies with control groups that did not meet our requirement, such as no control group or drug group control. (9) studies data errors or miss.

### 2.4 Literature screening and data extraction

According to the inclusion and exclusion criteria, two independent reviews (H.J. and C.Y.) conducting searches with a unified method and standardized search and select were used. Then the two researchers extracted the data and used it to create images and tables for reference. Any problems in data extraction are resolved by an independent adjudicator (R.M.).

The following data were collected: (1) basic information extracted, including the first author’s name and the year of publication; (2) the basic characteristics of the subjects, such as age and menopause years; (3) information of the study design, such as interventions, sample size, measurement parameters, duration of intervention and information related to bias risk assessment.

All the measurement parameters of BMD were covered to mean differences (MD) under the international standard system of units. If a study reported outcomes of different follow-up time or had more than one trial arm, then each different time and trial arm were treated as an independent trial. The Cochrane Handbook of Systematic Reviews of Interventions provides detailed measures to deal with this situation - one way to overcome this situation is to perform fixed-effects meta-analyses in within-study comparisons and random-effects meta-analyses between studies. In practice, the differences between the different analyses may be trivial (23).

Since the units of a given BMD parameter are not uniform, the standardized mean difference was chosen to account for the pooled effect. The software STATA^®^ 17(StataCorp LLC, College Station, TX, United States) was used to analyze the merger effect.

### 2.5 Quality assessment

The Cochrane Collaboration tool was used to assess the risk of bias. Two researchers (H.J. and C.Y.) independently assessed and graded the study quality according to the criteria described in the Cochrane Handbook. The risk of bias graph and the risk of bias summary graph were produced by RevMan5.4. If the evaluation results were inconsistent, the issue was resolved by consulted a third researcher (R.M).

### 2.6 Statistical analysis

We deal with the statistical analysis by used a set of multivariate meta-analysis programs in STATA 17 (Stata Crop LLC, College Station, TX, United States) software. We used this software draw the net relation diagram of different interventions, output the table of pairwise comparisons of the different interventions and the pairwise comparisons forest plot. When there was a closed loop, we should judge its direct and indirect comparison consistency by the node-splitting value, and when P<0.05 the inconsistency was considered to be significant. When there was no closed-loop structure within the different interventions, there was no need to make a consistence test. All outcome parameters were analyzed by mean differences (MD) and 95% credible intervals (95%CI). The surface under the cumulative ranking curve (SUCRA) was used to rank the effectiveness of six different interventions. SUCRA values range from 0 to 100%. The higher the SUCRA value, the higher the likelihood that the intervention effect is in the top rank or one of the top ranks.

## 3 Results

### 3.1 Literature selection

**Figure 1:**
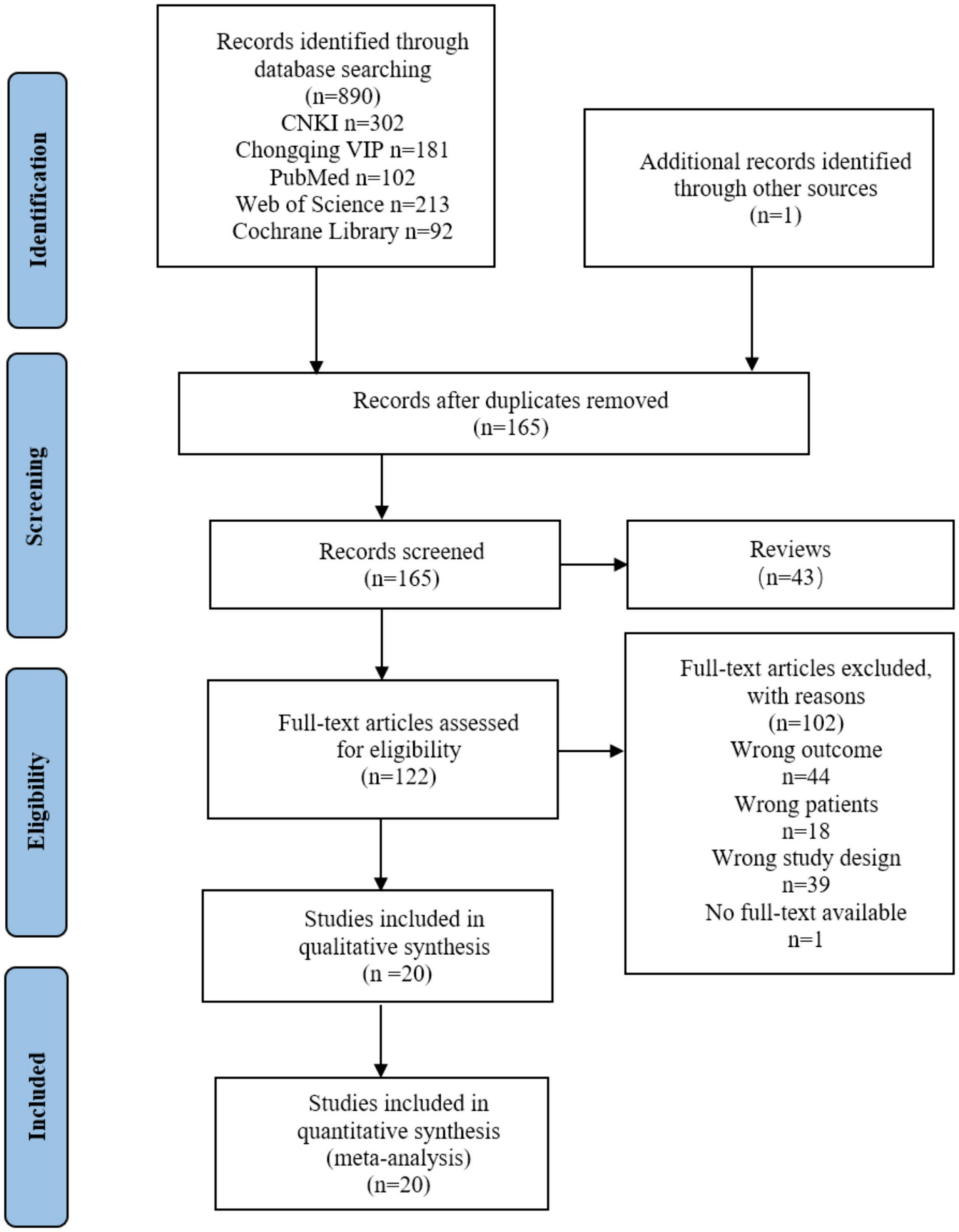
The PRISMA flow diagram of search and study selection.

In our initial search, we found a total of 890 articles, of which 726 were duplicates and were thus excluded. After removing the duplication and according to the exclusion and inclusion criteria, 20 studies out of the total 890 studies were selected for analysis. The flow diagram can be seen in Figure 1, Which the figure shows the selection process for the relevant studies. Based on the information from all the included full texts, a summary measure and the results of data collection of each included study can be seen in Table 1.

**Table 1:** The information about the included studies.

Study ID	Intervention	Sample	Age	Duration of infertility	Duration	Frequency	Outcomes
Zhou Yong, 2003	NEI	12	55.96 ± 2.8	6.35 ± 1.54	40 W	5-7times/week, 45-60 min/time	①
	TC	12	57.10 ± 2.71	7.12 ± 1.6			
	AE	12	56.78 ± 2.63	7.09 ± 1.58			
Wang Fuhong, 2018	WQX	25	65.6 ± 3. 8	NA	24 W	4times/week, 70 min/time	①②③④
	NEI	26	66.0 ± 4.4				
Zhou Yong, 2005	TC	16	57.21 ± 3.41	8.39 ± 2.47	24 W	5-7times/week, 45-60 min/time	①
	NEI	16					
	CA	16					
Ye chaoqun, 2016	TA	16		NA	24 W	3times/week, 30-60 min/time	②③
	TC	17	50–65				
	NEI	22					
Mao Hongni, 2009	TC	20	56.78 ± 2.91	6.78 ± 3.04	20 W	NA	①
	NEI	20					
	TA	20					
	CA	20					
Yu Dinghai, 2014(1)	TC	30	59.19 ± 3.64	NA	48 W	4times/week, 60 min/time	②④
	NEI	31	58.48 ± 3.45				
Yu Dinghai, 2014(2)	TC	34	57.29 ± 3.24	NA	48 W	4times/week, 60 min/time	②④
	NEI	31	58.48 ± 3.45				
Li Jingjing, 2019	YJJ	22	66.2 ± 3.5	NA	24w	5times/week, 70 min/time	①②③④
	WQX	22	65.7 ± 3.0				
	BDJ	21	65.5 ± 4.4				
	NEI	25	65.7 ± 2.9				
Shen Maorong, 2013	WQX	30	60.44 ± 6.11	10.07 ± 5.39	24 W	6times/week, 45 min/time	①
	NEI	30	60.07 ± 5.08	9.67 ± 4.94			
Zhou Yong, 2004(1)	TC	12	55.94 ± 2.83	6.58 ± 1.53	40 W	5-7times/week, 45-60 min/time	①
	NEI	12					
	AE	12					
Zhou Yong, 2004(2)	TC	12	55.94 ± 2.83	6.58 ± 1.53	40 W	5-7times/week, 45-60 min/time	①
	NEI	12					
	AE	12					
Li Yanfeng, 2021(1)	BDJ	36	65.2 ± 3.6	NA	12 W	5times/week, 60 min/time	①
	NEI	35	65.7 ± 3.7				
Li Yanfeng, 2021(2)	BDJ	36	65.2 ± 3.6	NA	24 W	5times/week, 60 min/time	①
	NEI	35	65.7 ± 3.7				
Cai Yingxian, 2018	BA	30	51.4 ± 4.9	NA	12 W	NA	①
	CA	30	52.1 ± 4.2				
Song Jinglin, 2018	TC	28	64.3 ± 3.2	NA	48w	5times/week, 70 min/time	①②③④
	NEI	31	64.7 ± 4.1				
	AE	29	64.8 ± 2.9				
Li Jingya, 2017	TC	36	64.4 ± 3.5	NA	48w	1times/week, 60 min/time	①②③④
	NEI	37	65.2 ± 3.7				
Li Jingya, 2017(1)	TC	37	64.8 ± 4.1	NA	48w	3times/week, 60 min/time	①②③④
	NEI	37	65.2 ± 3.7				
Li Jingya, 2017(2)	TC	38	65.2 ± 3.7	NA	48w	6times/week, 60 min/time	①②③④
	NEI	37	65.2 ± 3.7				
Xu Fei, 2017	TC	43	56.2 ± 5.6	7.4 ± 2.7	24 W	6-7times/week, >45 min/time	①②③④
	NEI	43	57.1 ± 6.0	7.6 ± 2.9			
Xu Fei, 2017(1)	TC	43	56.2 ± 5.6	7.4 ± 2.7	48 W	6-7times/week, >45 min/time	①②③④
	NEI	43	57.1 ± 6.0	7.6 ± 2.9			
Huiru Wang, 2015	TC	34	58.54 ± 3.37	NA	48 W	4times/week, 60 min/time	①②③
	NEI	35	58.54 ± 3.37				
Huiru Wang, 2015(1)	TC	37	57.93 ± 3.22	NA	48 W	4times/week, 60 min/time	①②③
	NEI	35	58.54 ± 3.37				
Peter M Wayne, 2012	NEI	43	60.4 ± 5.3	NA	36 W	4times/week, 30 min/time	①
	TC	26	59.1 ± 4.9				
Liang Cheng, 2020	NEI	17	61.9 ± 2.5	NA	48 W	5times/week, 30 min/time	①②③④
	TC	17	61.3 ± 2.4				
Liang Cheng, 2020(1)	NEI	17	61.9 ± 2.5	NA	48 W	5times/week, 60 min/time	①②③④
	TC	18	61.5 ± 3.0				
Kaiming Chan, 2004	TC	54	54.4 ± 3.3	4.9 ± 2.5	48 W	5times/week, 50 min/time	①③④
	NEI	49	53.6 ± 3.2	4.5 ± 2.4			
Song, R., 2010	TC	30	63.03 ± 7.27	NA	24 W	7times/week, 45-60 min/time	②③④
	NEI	35	61.20 ± 7.96				
Liu Bao-Xin, 2015	BDJ	48	61.87 ± 8.29	13.24 ± 6.77	48 W	3times/day, 7repetitions/time	①③
	NEI	42	63.23 ± 7.56	13.79 ± 6.27			
	CA	45	62.29 ± 6.47	12.53 ± 5.69			
	BA	49	61.45 ± 5.89	11.21 ± 5.29			

TC, Tai chi; BDJ, Baduanjin; YJJ, Yijinjing; WQX, Wuqingxi; TA, Tai chi plus calcium; BA, Baduanjin plus calcium; NEI, Not exercise intervention; CA, Calcium supplement; AE, Aerobic exercise; NA, not available; ①, Lumbar L2-L4 bone mineral density; ②, Femur ward’s triangle bone mineral density; ③, Femur neck bone mineral density; ④, Femur greater trochanter bone mineral density.

### 3.2 Characteristics of the included studies and results of risk of bias

This review included 20 studies involving 1933 subjects (24–43). All subjects were femes with 45 or older. Six traditional Chinese exercise interventions (TC, BDJ, YJJ, WQX, TA, BA) were included in the current review. The control groups (NEI, CA, AE) were included. There were 20 studies varied widely in duration from the shortest 12 weeks to the longest 48 weeks. Among the included studies, 14 studies compared TC with NEI (25, 26, 28, 31, 33, 34, 36–43), 3 studies compared TC with AE (33, 41, 42), 2 studies compared TC with CA (31, 43), 3 studies compared BDJ with NEI (27, 29, 30), 1 studies compared BDJ with CA (30), 1 studies compared YJJ with NEI (27), 3 studies compared WQX with NEI (27, 32, 35), 2 studies compared TA with NEI (31, 43), 2 studies compared TA with CA (31, 43), 1 study compared BA with NEI (30), 2 study compared BA with CA (24, 30). The outcome indicators were the bone density of lumbar L2-L4, femur ward’s triangle, femur neck, and femur greater trochanter. None of the 20 studies reported any adverse reactions. The basic information of the 20 research literature included in the study is shown in Table 1.

**Figure 2:**
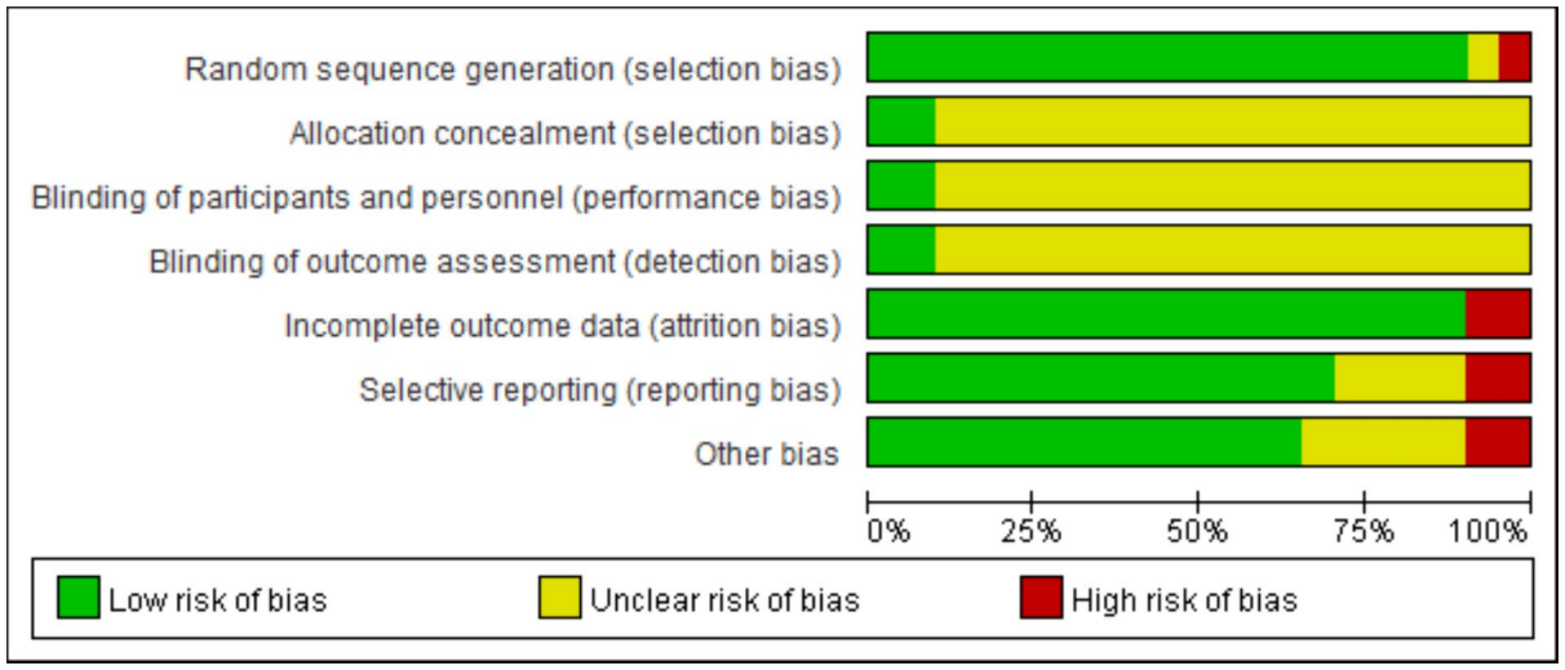
Risk of bias graph for the included RCTs using the bias risk assessment tool recommended in the Cochrane5.1 version of the system review manual.

The risk of bias in the 20 included studies was assessed, and the consensus was reached after discussion. The overall result is shown in Figure 2. Of the 20 RCTs, 18 studies did not mention whether the allocation was hidden (24–28, 30–33, 35–43), 1 studies mentioned whether the researchers and subjects were double-blinded (34), and only 10 percentage of the studies describe the participant or the staff and the evaluator was blind (33, 37). Only two studies had incomplete results due to subjects dropping out and the other did not fully account for them (29, 36). All studies recorded their research plans and researched according to the plans.

As the intervention methods included in this study were all exercise therapy, the implementation of the blind methods (subject, staff and outcome assessor) had little influence on the results, so most of the included RCTs did not adapt blind methods. In addition, other bias was mainly low risk bias, so the overall quality of the included literature was high.

### 3.3 The results of the network meta-analysis

#### 3.3.1 Evidence network relationship

**Figure 3:**
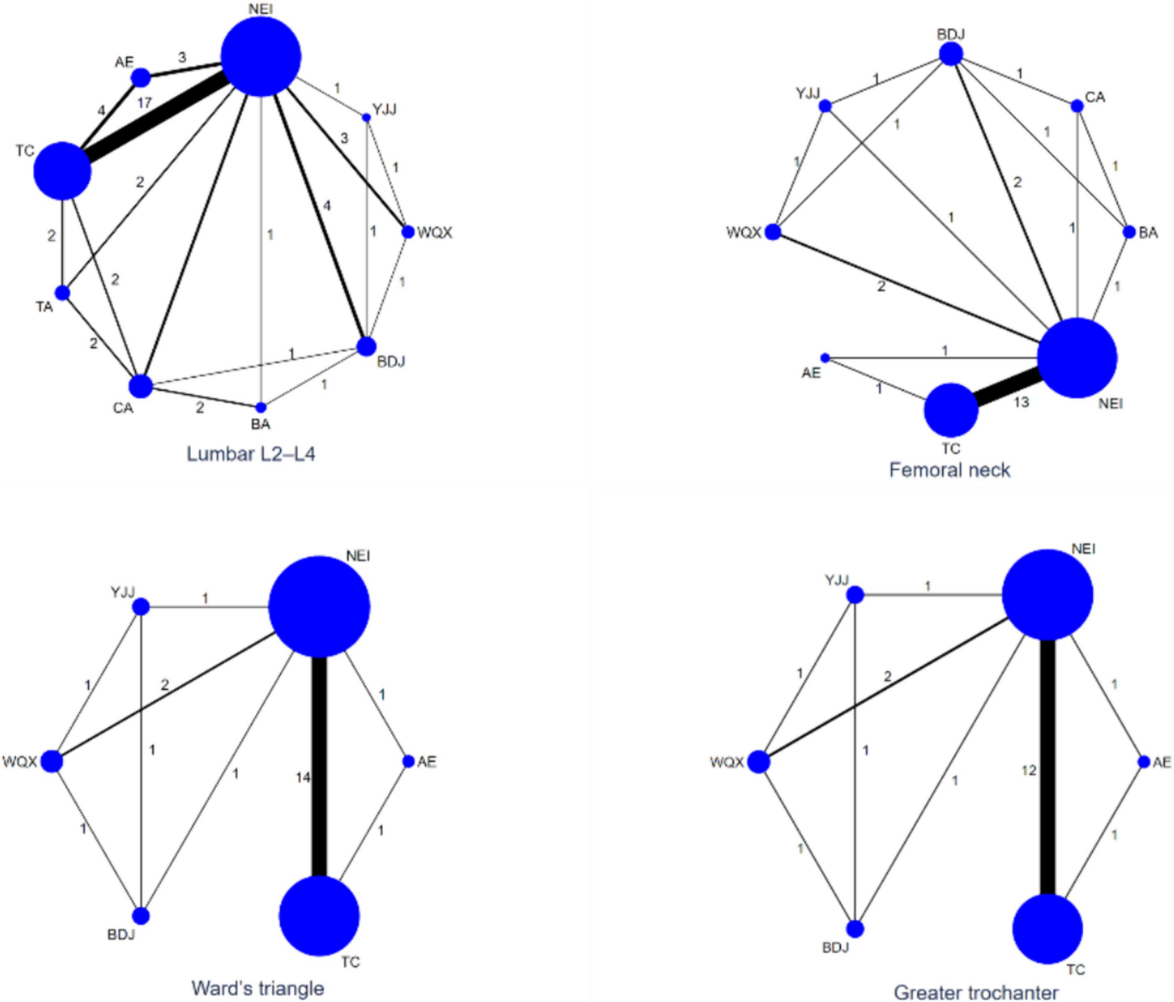
Evidence network diagram of network meta-analysis. TC, Tai chi; BDJ, Baduanjin; YJJ, Yijinjing; WQX, Wuqingxi; TA, Tai chi plus calcium; BA, Baduanjin plus calcium; NEI, Not exercise intervention; CA, Calcium supplement; AE, Aerobic exercise. The area of the circle, the size of the corresponding intervention study sample size; Nodes represent treatment or intervention; Lines show where direct comparisons exist from one or more studies.

The line connecting the net point indicate the direct comparison evidence between networks. The thickness of the line between the nodes indicates the number of experiments between the different interventions represented by the different nodes. In the network diagram of the effects of nine interventions (concluding the control group) on bone mineral density in postmenopausal women (Figure 3), the area of the nodes represents the size of the corresponding intervention study sample size. The sample sizes for all nine interventions for four different outcomes are shown in Figure 3.

#### 3.3.2 Lumbar L2–L4 BMD

Using the classical frequency method with Stata software 17, simultaneously all intervention measures are evaluated through a combination of traditional direct and indirect comparisons. A summary graph of the network meta-analysis is presented in Table 2.

**Table 2:** Comparative analysis of total effective rate.

Items		Lumbar L2–L4	Femoral neck	Ward’s triangle	Greater trochanter
TC	YJJ	−0.26 (−1.62,1.10)	0.66 (−1.14,2.46)	1.15 (−0.80,3.09)	0.27 (−2.11,2.65)
	WQX	0.49 (−0.43,1.42)	0.29 (−1.13,1.71)	0.59 (−0.92,2.10)	0.63 (−1.22,2.49)
	BDJ	−1.13 (−1.94,-0.32)*	1.20 (−0.20,2.61)	0.76 (−1.18,2.71)	0.78 (−1.61,3.16)
	TA	−0.13 (−1.09,0.83)			
	BA	−1.25 (−2.36,-0.14)*	−2.53 (−4.40,-0.66)*		
	AE	0.47 (−0.25,1.18)	1.51 (−0.16,3.18)	1.18 (−0.55,2.92)	1.08 (−1.04,3.21)
	CA	0.31 (−0.48,1.11)	1.23 (−0.60,3.06)		
	NEI	0.99 (0.62,1.37)*	1.24 (0.70,1.78)*	1.63 (1.09,2.17)*	0.98 (0.28,1.68)*
	WQX	0.76 (−0.62,2.14)	−0.37 (−2.16,1.42)	−0.56 (−2.42,1.31)	0.36 (−1.91,2.64)
	BDJ	−0.87 (−2.22,0.49)	0.55 (−1.24,2.34)	−0.38 (−2.38,1.62)	0.51 (−1.93,2.95)
	TA	0.13 (−1.48,1.74)			
YJJ	BA	−0.98 (−2.64,0.67)	−3.19 (−5.52,-0.86)*		
	AE	0.73 (−0.77,2.22)	0.85 (−1.54,3.25)	0.04 (−2.51,2.58)	0.81 (−2.30,3.93)
	CA	0.57 (−0.92,2.06)	0.57 (−1.73,2.87)		
	NEI	1.25 (−0.06,2.56)	0.58 (−1.13,2.30)	0.48 (−1.38,2.35)	0.71 (−1.56,2.99)
WQX	BDJ	−1.62 (−2.65,-0.60)*	0.92 (−0.65,2.48)	0.17 (−1.70,2.04)	0.14 (−2.14,2.42)
	TA	−0.63 (−1.89,0.64)			
	BA	−1.74 (−3.08,-0.40)*	−2.82 (−4.93,-0.71)*		
	AE	−0.03 (−1.13,1.08)	1.22 (−0.91,3.34)	0.59 (−1.64,2.83)	0.45 (−2.28,3.18)
	CA	−0.18 (−1.30,0.94)	0.94 (−1.13,3.02)		
	NEI	0.50 (−0.35,1.34)	0.95 (−0.36,2.26)	1.04 (−0.38,2.45)	0.35 (−1.37,2.07)
BDJ	TA	0.99 (−0.17,2.16)			
	BA	−0.12 (−1.27,1.03)	−3.74 (−5.53,-1.95)*		
	AE	1.60 (0.58,2.61)*	0.30 (−1.81,2.42)	0.42 (−2.13,2.97)	0.31 (−2.81,3.42)
	CA	1.44 (0.49,2.38)*	0.02 (−1.72,1.77)		
	NEI	2.12 (1.39,2.85)*	0.03 (−1.26,1.33)	0.87 (−1.00,2.73)	0.20 (−2.07,2.48)
TA	BA	−1.11 (−2.45,0.22)			
	AE	0.60 (−0.56,1.76)			
	CA	0.44 (−0.59,1.48)			
	NEI	1.12 (0.17,2.07)*			
BA	AE	1.71 (0.44,2.99)*	4.04 (1.59,6.49)*		
	CA	1.56 (0.56,2.55)*	3.76 (1.85,5.68)*		
	NEI	2.24 (1.16,3.31)*	3.77 (1.98,5.56)*		
AE	CA	0.16 (−0.87,1.18)	−0.28 (−2.70,2.14)		
	NEI	0.52 (−0.19,1.24)	−0.27 (−1.94,1.40)	0.44 (−1.29,2.18)	−0.10 (−2.23,2.02)
CA	NEI	0.68 (−0.08,1.44)	0.01 (−1.74,1.76)		

TC, Tai chi; BDJ, Baduanjin; YJJ, Yijinjing; WQX, Wuqingxi; TA, Tai chi plus calcium; BA, Baduanjin plus calcium; NEI, Not exercise intervention; CA, Calcium supplement; AE, Aerobic exercise; The data represent the comparison of outcomes between two interventions. *Data are standardized mean difference (95% CI) of combined effect, *p* < 0.05.

As shown in Figure 3, a total of 24 studies involving 1716 subjects reported lumbar L2–L4 BMD levels. Compared with the NEI group, the TC [MD = 0.99 (0.62, 1.37)], the BDJ [MD = 2.12 (1.39, 2.85)], the TA [MD = 1.12 (0.17, 2.07)] and the BA [MD = 2.24 (1.16, 3.31)] groups were superior improved the level of lumbar L2–L4 BMD (*p* < 0.05). Compared with the CA group, the BDJ [MD = 1.44 (0.49, 2.38)] and the BA [MD = 1.56 (0.56, 2.55)] groups were significantly improved the level of Lumbar L2–L4 BMD (*p* < 0.05). Compared with the AE group, the BDJ [MD = 1.60 (0.58, 2.61)] and the BA [MD = 1.71 (0.44, 2.99)] groups were significantly improved the level of Lumbar L2–L4 BMD (*p* < 0.05). Compared with the BDJ and the BA groups, the TC [MD = 1.13 (0.32, 1.94), MD = 1.25 (0.14, 2.36)] and the WQX [MD = 1.62 (0.60, 2.65), MD = 1.74 (0.40, 3.08)] groups were more effective improved Lumbar L2–L4 BMD (*p* < 0.05). There was no statistical significance in the other groups (Table 2).

#### 3.3.3 Femoral neck BMD

In terms of femoral neck BMD, there were 16 studies involving 1,241 subjects were analyzed (Figure 3). Compared with the NEI group, the TC [MD = 1.24 (0.7, 1.78)] and the BA [MD = 3.77 (1.98, 5.56)] groups were superior improved the femoral neck BMD level (*p* < 0.05). Compared with the CA group, the BA [MD = 3.76 (1.85, 5.68)] group was significantly improved the femoral neck BMD level (*p* < 0.05). Compared with the AE group, the BA [MD = 4.04 (1.59, 6.49)] group was significantly improved the femoral neck BMD level (*p* < 0.05). Compared with the BA group, the TC [MD = 2.53 (0.66, 4.40)], the YJJ [MD = 3.19 (0.86, 5.52)], the WQX [MD = 2.82 (0.71, 4.93)] and the BDJ [MD = 3.74 (1.95, 5.53)] group were more effective improved the femoral neck BMD level (*p* < 0.05). There was no statistical significance in the other groups (Table 2).

#### 3.3.4 Ward’s triangle BMD

Regarding Ward’s triangle BMD, a total of 16 studies analyzed 1,080 subjects (Figure 3). Compared with the NEI group, the TC [MD = 1.63 (1.09, 2.17)] was superior improved the Ward’s triangle BMD level (*p* < 0.05). There was no statistical significance in the other groups (Table 2).

#### 3.3.5 Greater trochanter BMD

In terms of Greater trochanter BMD, there were 14 studies involving 986 subjects (Figure 3). Compared with the NEI group, the TC group [MD = 0.98 (0.28, 1.68)] was superior to increasing the level of greater trochanter BMD (*p* < 0.05). There was no statistical significance in the other groups (Table 2).

### 3.4 Intervention ranking

Table 3 shows the cumulative probability data of each intervention, the ranking probability gram can help researchers to predict the best or worst intervention quickly, but interventions with higher ranking probability are not necessarily the best effective, there are still many undetermined factors that can affect the ranking. If the best intervention is not available, the SUCRA probability gram able to help in decision making.

**Table 3:** A network league table based on the network meta-analysis from data.

Treatments/Outcomes	SUCRA	PrBest	MeanRank
Lumbar L2–L4			
BA	92.2	54.4	1.6
BDJ	90.9	38.3	1.7
YJJ	60.6	5.8	4.2
TA	57.5	1.4	4.4
TC	53.8	0	4.7
CA	35.7	0	6.1
AE	28.3	0	6.7
WQX	27.6	0	6.8
NEI	3.5	0	8.7
Femoral neck			
BA	99.8	98.8	1
TC	74.9	0.4	2.8
WQX	64	0.5	3.5
YJJ	50.4	0.3	4.5
CA	30.5	0	5.9
BDJ	29.9	0	5.9
NEI	28	0	6
AE	22.5	0	6.4
Ward’s triangle			
TC	86.9	55.9	1.7
WQX	63.2	15.1	2.8
BDJ	54.2	15.7	3.3
YJJ	39.3	6.8	4
AE	38.7	6.4	4.1
NEI	17.7	0	5.1
Trochanter			
TC	77.7	36.8	2.1
YJJ	62.5	29.2	2.9
WQX	49.6	10.4	3.5
BDJ	44.1	13.3	3.8
AE	34.5	10.2	4.3
NEI	31.5	0	4.4

TC, Tai chi; BDJ, Baduanjin; YJJ, Yijinjing; WQX, Wuqingxi; TA, Tai chi plus calcium; BA, Baduanjin plus calcium; NEI, No exercise intervention; CA, Calcium supplement; AE, Aerobic exercise; SUCRA, the surface under the cumulative ranking; PrBest, the probability that the treatment becomes the best treatment; Mean Rank, the ranking of the treatment measures.

**Figure 4:**
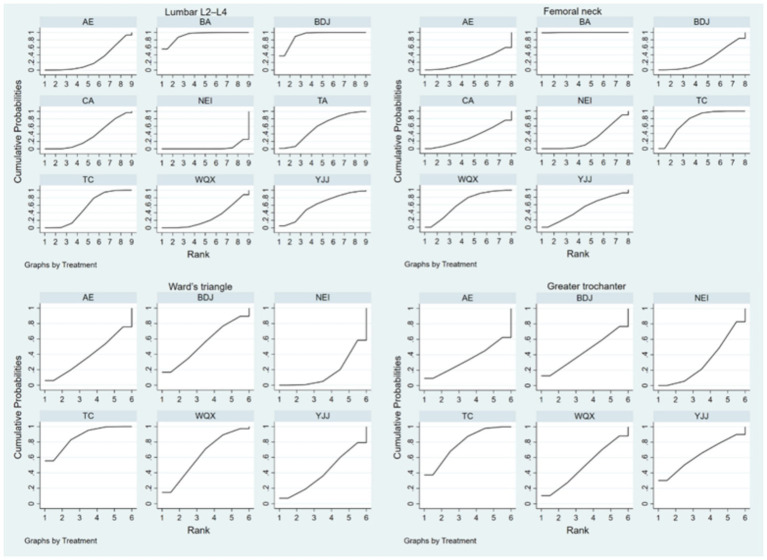
The surface under the cumulative ranking plots based on the cumulative probabilities of the interventions. TC, Tai chi; BDJ, Baduanjin; YJJ, Yijinjing; WQX, Wuqingxi; TA, Tai chi plus calcium; BA, Baduanjin plus calcium; NEI, Not exercise intervention; CA, Calcium supplement; AE, Aerobic exercise.

Based on the included studies, the cumulative probability being the best intervention to lumbar L2–L4 BMD was 92, 90.9, 60.8, 57.9, 53.5, 35.2, 28.2, 27.9, and 3.5% for BA, BDJ, YJJ, TA, TC, CA, WQX, AE, and NEI, respectively. The cumulative probability being the best intervention to femoral neck BMD was 99.8, 74.9, 64, 50.4, 30.5, 29.9, 28, and 22.5% for BA, TC, WQX, YJJ, CA, BDJ, NEI, and AE, respectively. The cumulative probability being the best intervention to Ward’s Triangle BMD was 96.4, 76.7, 55.6, 39.2, 29.8, and 2.2% for TC, YJJ, WQX, BDJ, AE, and NEI, respectively. The cumulative probability being the best intervention to greater trochanter BMD was 77.7, 62.5, 49.6, 44.1, 34.5, and 31.5% for TC, YJJ, WQX, BDJ, AE, and NEI, respectively (Figure 4; Table 3).

### 3.5 Consistency analysis

Consistency can be evaluated by node splitting, with each direct comparison being excluded from the network and then estimating the difference between this direct evidence and the indirect evidence from the network. Conversely, if there is difference, it means that there are inconsistencies, which need to be fitted with an inconsistent model. If there are no differences, it means that there are no inconsistencies, and the consistency model is used to fit it.

As can be seen from Table 4, all *p*-values were greater than 0.05, indicating that there was no obvious inconsistency.

**Table 4:** Inconsistency test between direct and indirect treatment comparisons in mixed treatment comparison.

Side	Direct		Indirect		Difference		
	Coef.	Std. Err.	Coef.	Std. Err.	Coef.	Std. Err.	P > |z|
Lumbar L2–L4							
AE	−0.21	0.57	0.11	1.01	−0.32	1.16	0.78
AG	0.27	0.41	1.30	0.83	−1.03	0.93	0.27
AH	0.33	0.57	0.29	0.60	0.04	0.83	0.96
AI	0.98	0.20	1.75	1.60	−0.77	1.61	0.63
BC	0.98	0.79	−0.18	1.62	1.16	1.80	0.52
BD	−1.43	0.77	1.08	1.46	−2.51	1.67	0.13
BI	1.56	0.79	0.42	1.32	1.14	1.55	0.46
CD	−2.41	0.79	−1.04	0.68	−1.37	1.05	0.19
CI	0.67	0.43	−1.84	1.61	2.51	1.67	0.13
DF	−0.46	0.76	0.43	0.95	−0.89	1.22	0.46
DH	1.37	0.77	1.49	0.64	−0.12	1.01	0.91
DI	2.11	0.40	2.20	1.18	−0.09	1.25	0.94
EH	0.54	0.57	−0.24	1.52	0.78	1.63	0.63
EI	0.95	0.57	1.62	0.97	−0.68	1.12	0.55
FH	1.47	0.55	2.25	1.53	−0.78	1.63	0.63
FI	2.52	0.77	1.93	0.80	0.59	1.12	0.60
GI	0.32	0.41	1.35	0.83	−1.03	0.93	0.27
HI	0.50	0.46	1.19	0.78	−0.69	0.91	0.45
Femoral neck							
AG	0.88	0.94	3.59	1.71	−2.71	1.95	0.17
AI	1.24	0.28	0.62	18.26	0.62	18.26	0.97
BC	−0.20	1.02	−1.47	2.60	1.27	2.80	0.65
BD	0.48	1.03	0.97	2.59	−0.49	2.79	0.86
BI	0.48	1.03	0.99	2.05	−0.51	2.29	0.82
CD	0.68	1.03	1.38	1.43	−0.70	1.76	0.69
CI	0.98	0.73	0.50	2.69	0.48	2.79	0.86
DF	−3.80	1.03	−3.31	2.61	−0.49	2.79	0.86
DH	−0.04	1.01	0.45	2.60	−0.49	2.79	0.86
DI	−0.05	0.71	1.22	2.70	−1.27	2.79	0.65
FI	3.71	1.03	4.20	2.61	−0.49	2.79	0.86
GI	−0.89	0.94	1.81	1.72	−2.71	1.95	0.17
HI	−0.05	1.01	0.43	2.60	−0.49	2.79	0.86
Ward’s triangle							
AF	0.62	0.41	2.61	0.74	−1.99	0.84	0.02
AI	0.73	0.13	0.54	22.36	0.19	22.36	0.99
BC	−0.38	0.49	−1.76	1.30	1.38	1.40	0.33
BD	0.31	0.49	1.69	1.31	−1.37	1.40	0.33
BI	0.31	0.49	1.69	1.31	−1.37	1.40	0.33
CD	0.00	0.49	1.38	1.31	−1.38	1.40	0.33
CI	1.03	0.35	1.41	44.72	−0.38	44.72	0.99
DI	0.69	0.49	2.07	1.31	−1.37	1.40	0.33
EI	−0.82	0.41	1.17	0.74	−1.99	0.84	0.02
Trochanter							
AF	0.56	1.25	2.84	2.29	−2.28	2.61	0.38
AI	0.98	0.36	0.24	22.38	0.74	22.38	0.97
BC	0.47	1.30	−0.35	3.42	0.82	3.66	0.82
BI	0.61	1.30	1.43	3.42	−0.82	3.66	0.82
CD	0.04	1.30	0.86	3.42	−0.82	3.66	0.82
CI	0.35	0.88	1.83	44.75	−1.48	44.76	0.97
DI	0.10	1.30	0.93	3.42	−0.82	3.66	0.82
FI	−0.62	1.25	1.66	2.29	−2.28	2.61	0.38

Coef., coefficient; Std. Err., standard error; A, TC (Tai chi); B, YJJ (Yijinjing); C, WQX (Wuqingxi); D, BDJ (Baduanjin); E, TA (Tai chi plus calcium); F, BA (Baduanjin plus calcium); G, AE (Aerobic exercise); H, CA (Calcium supplement); I, NEI (Not exercise intervention); p>0.05, there is no inconsistency.

## 4 Discussion

In recent years, TCEs has been widely used at home and abroad to prevent osteoporosis. As an important component of non-drug therapy, traditional Chinese exercise has many benefits for postmenopausal women, which can not only effectively enhance muscle strength, improve balance ability, reduce the risk of falls, and reduce the incidence of fractures caused by osteoporosis. And because this exercise method is mostly carried out outdoors, it can increase the conversion of vitamin D and promote the absorption of calcium through sunlight. In addition, it is easy to operate, economical and safe, so it can quickly alleviate bone loss and improve the quality of life of patients with bone loss (44). This study was written based on the hypothesis II that TCEs have a positive effect on BMD in postmenopausal women. This systematic review included 20 randomized controlled trials with a total of 6 TCEs, including 1933 subjects, all aged over 45 years, providing high-quality evidence for the effects of the six TCEs on BMD. Yu Ying studied the improvement of osteoporosis by four TCEs in middle-aged and older adult people through network meta-analysis but did not analyze this group of postmenopausal women separately (45). To the best of our knowledge, at present, this review is the first network meta-analysis comparing the effects of multiple TCEs.

In this network meta-analysis, we included 20 studies that investigated the effect of TCEs on BMD at various sites in postmenopausal women. These studies included a total of six interventions and three general controls. Direct and indirect evidence from network Meta-analysis showed that TC, BDJ, TA and BA interventions could partially improve bone mineral density in postmenopausal women. Compared with NEI, TC significantly improved BMD of lumbar spine, femoral neck, Ward’s triangle, and greater trochanter of femur in postmenopausal women. BDJ significantly improved BMD of the lumbar spine in postmenopausal women. TA significantly improved BMD of the lumbar spine in postmenopausal women. BA significantly improved BMD at the lumbar spine and femoral neck in postmenopausal women. We can conclude that the above four interventions have a significant impact on the improvement of some indicators of bone mineral density in postmenopausal women. The consistency analysis showed no significant differences, indicating that the statistical model for indirect comparisons was reliable. This may be due to similar exercise programs and outcome measures in the included studies. Therefore, postmenopausal women can be advised to choose an appropriate TCE according to their preferences and physical conditions to avoid a decrease in BMD.

The ranking of lumbar L2-L4 BMD was BA > BDJ > YJJ > TA > TC > CA > WQX > AE > NEI. Compared with NEI, BA, BDJ, TA and TC showed significant improvement, among which BA had the best effect on improving lumbar BMD. The ranking of femoral neck BMD was BA > TC > WQX > YJJ > CA > BDJ > NEI > AE. Compared with NEI, BA and TC showed significant improvement, and BA had the best effect on improving femoral neck BMD. The rank of Ward’s triangle BMD was TC > WQX > BDJ > YJJ > AE > NEI. Compared with the control group, TC had a significant and best effect on improving Ward’s triangle BMD. The ranking of femoral greater trochanter BMD was TC > YJJ > WQX > BDJ > AE > NEI. Compared with NEI, TC had the most significant and best effect on improving femoral greater trochanter BMD. In addition, statistical factors such as study design, number of controls, number of subjects, and many other factors may contribute to differences in results. Although the main motor patterns of different types of TCE are similar, their activity patterns and training principles are different.

In conclusion, TC and BA were found to be more effective in improving BMD in postmenopausal women compared with NEI, AE and CA groups, and TC was significantly effective on all four indicators of BMD. However, BA was only effective for two BMD indices, lumbar L2-L4 and femoral neck. Therefore, TC is recommended as a suitable TCE method for improving BMD in postmenopausal women.

The mechanism of TC on bone mineral density in postmenopausal women is to continuously adjust the position of the body’s center of gravity to maintain balance and emphasize breathing during exercise. Long-term exercise increases the stress of the waist and lower limbs muscles of the subjects, thereby promoting the increase of bone mass and affecting bone mineral density (46). Previous studies have shown that calcium supplementation may increase BMD at the lumbar spine and femoral neck in postmenopausal women (47). Tai chi improves balance and reduces risk factors for fractures in the older adults (48, 49). It is reasonable to believe that there may be potential benefits of combining TCE and calcium supplementation. Therefore, TA, BA and CA groups were included in the study. We found that CA group did not show more significant improvement in BMD in postmenopausal women compared with NEI group. It is worth noting that both TA and BA groups have shown a more significant improvement in bone mineral density in postmenopausal women, but the research literature on TA and BA is limited, and future research can focus on this aspect.

## 5 Limitations of the study

This study has some innovations, but also some shortcomings. (1) Most of the studies were from China and published in Chinese, which may affect the results. (2) Most RCTs did not explicitly report whether they were randomized or double-blind. The timing of adaptation, scale and actual needs of each TCE were not discussed in this study. (3) In this study, CA and AE groups were included as control groups.

## 6 Conclusion

Our network meta-analysis demonstrated that four TCE (TC, BDJ, TA and BA) are all effective in partially enhancing BMD indicators in postmenopausal women, while TC was effective on all four BMD indicators, which seems to be recommended as the most suitable exercise modality for postmenopausal women. However, due to the limitations of this study, follow-up studies need to classify and explore the specific bone conditions of postmenopausal women, and further study the effects of exercise duration and frequency on bone mineral density parameters. In addition, future high-quality clinical trials are needed to strengthen the supporting evidence.

## Data Availability

The datasets presented in this study can be found in online repositories. The names of the repository/repositories and accession number(s) can be found at: Corpus ID: 267761979.
