# Supplementary material for: The effect of different traditional Chinese exercises on bone mineral density in menopausal women: a systematic review and network meta-analysis
Source: Front Public Health. 2024 Sep 12;12:1430608. doi: 10.3389/fpubh.2024.1430608 (PMC11425856; doi:10.3389/fpubh.2024.1430608)
Supplement: Supplementary file 1 [file Data_Sheet_1.docx]

**Appendix**


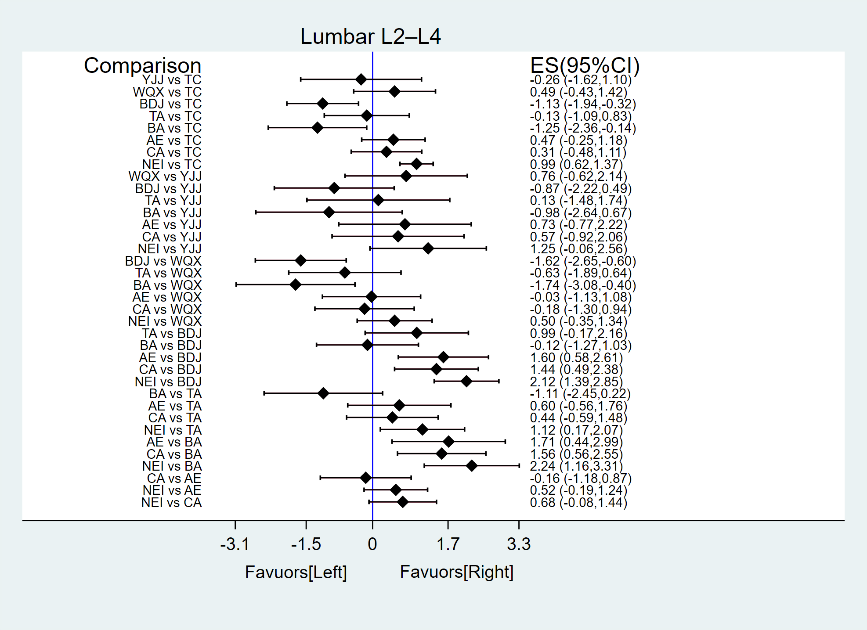


Figure A1. Forest plot of BMD of Lumbar L2-L4. Notes: Notes: TC, Tai chi; BDJ, Baduanjin; YJJ, Yijinjing; WQX, Wuqingxi; TA, Tai chi plus calcium; BA, Baduanjin plus calcium; NEI, Not exercise intervention; CA, Calcium supplement; AE, Aerobic exercise; ES, effect size.


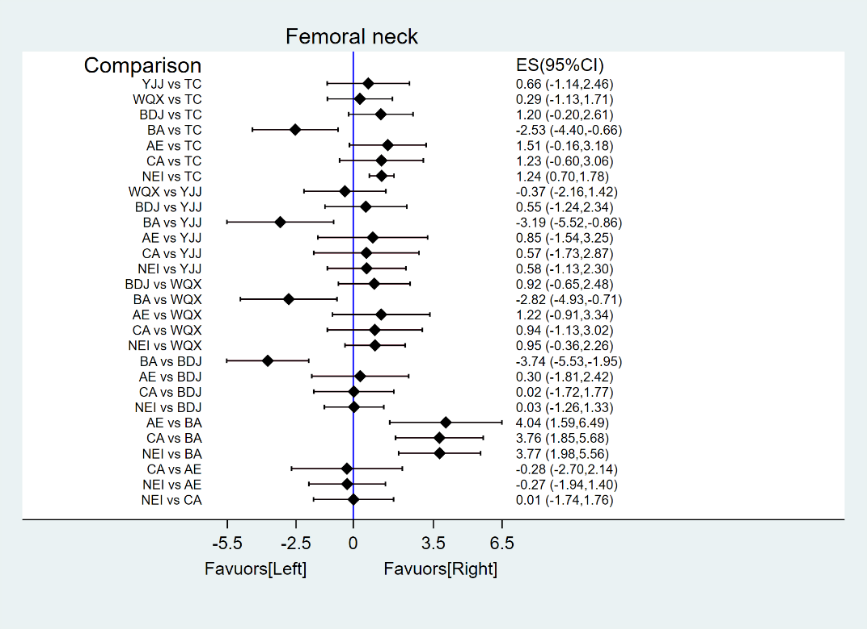


Figure A2. Forest plot of BMD of Femoral neck. Notes: Notes: TC, Tai chi; BDJ, Baduanjin; YJJ, Yijinjing; WQX, Wuqingxi; BA, Baduanjin plus calcium; NEI, Not exercise intervention; CA, Calcium supplement; AE, Aerobic exercise; ES, effect size.


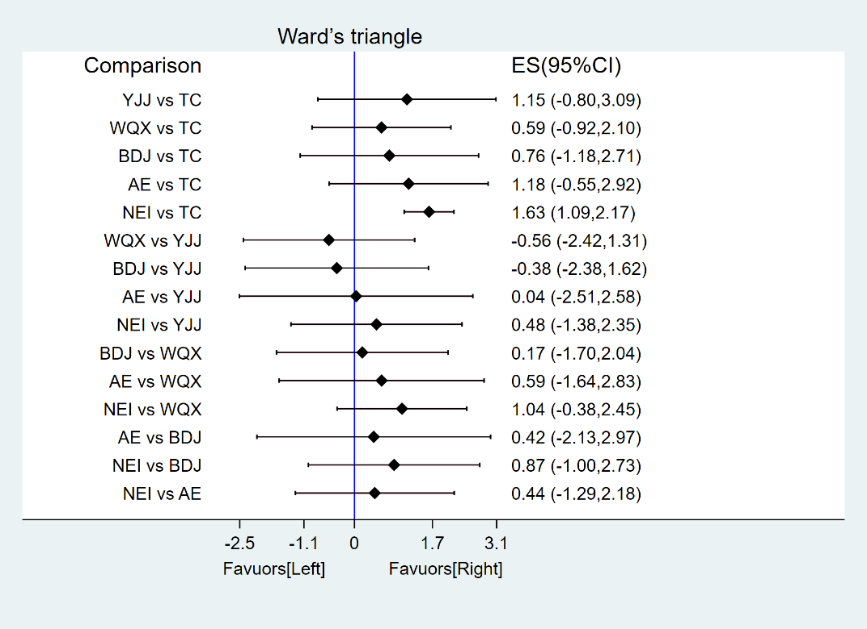


Figure A3. Forest plot of BMD of Ward’s triangle. Notes: Notes: TC, Tai chi; BDJ, Baduanjin; YJJ, Yijinjing; WQX, Wuqingxi; NEI, Not exercise intervention; AE, Aerobic exercise; ES, effect size.


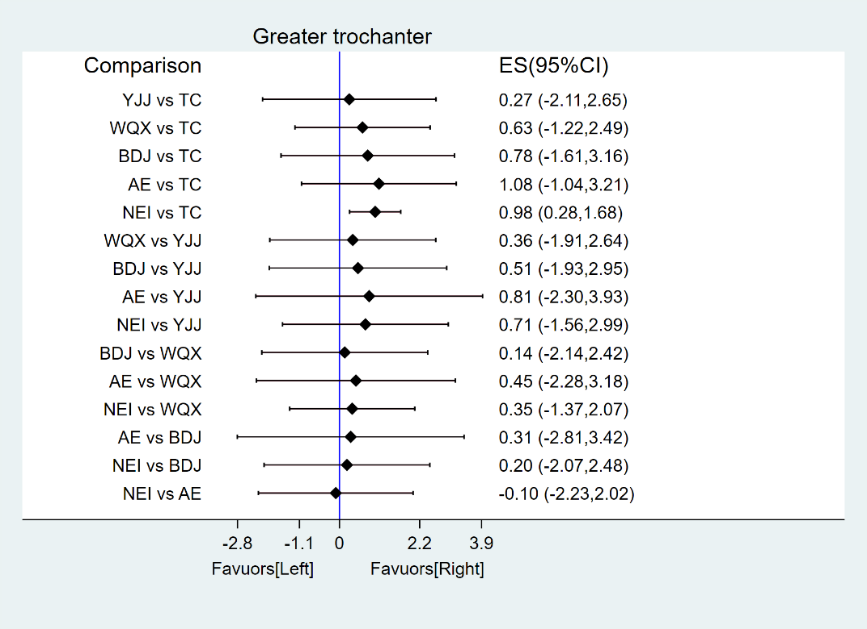


Figure A4. Forest plot of BMD of Greater trochanter. Notes: Notes: TC, Tai chi; BDJ, Baduanjin; YJJ, Yijinjing; WQX, Wuqingxi; NEI, Not exercise intervention; AE, Aerobic exercise; ES, effect size.
